# An analysis of potential barriers and enablers to regulating the television marketing of unhealthy foods to children at the state government level in Australia

**DOI:** 10.1186/1471-2458-12-1123

**Published:** 2012-12-28

**Authors:** Alexandra Chung, Jane Shill, Boyd Swinburn, Helen Mavoa, Mark Lawrence, Bebe Loff, Bradley Crammond, Gary Sacks, Steven Allender, Anna Peeters

**Affiliations:** 1 Department of Epidemiology and Preventive Medicine, BakerIDI Heart and Diabetes Institute, Monash University, Victoria, Australia; 2 WHO Collaborating Centre for Obesity Prevention, Deakin University, Victoria, Australia

**Keywords:** Unhealthy food, Regulation, Government, Children, Marketing, Advertising

## Abstract

**Background:**

In Australia there have been many calls for government action to halt the effects of unhealthy food marketing on children's health, yet implementation has not occurred. The attitudes of those involved in the policy-making process towards regulatory intervention governing unhealthy food marketing are not well understood. The objective of this research was to understand the perceptions of senior representatives from Australian state and territory governments, statutory authorities and non-government organisations regarding the feasibility of state-level government regulation of television marketing of unhealthy food to children in Australia.

**Method:**

Data from in-depth semi-structured interviews with senior representatives from state and territory government departments, statutory authorities and non-government organisations (n=22) were analysed to determine participants' views about regulation of television marketing of unhealthy food to children at the state government level. Data were analysed using content and thematic analyses.

**Results:**

Regulation of television marketing of unhealthy food to children was supported as a strategy for obesity prevention. Barriers to implementing regulation at the state level were: the perception that regulation of television advertising is a Commonwealth, not state/territory, responsibility; the power of the food industry and; the need for clear evidence that demonstrates the effectiveness of regulation. Evidence of community support for regulation was also cited as an important factor in determining feasibility.

**Conclusions:**

The regulation of unhealthy food marketing to children is perceived to be a feasible strategy for obesity prevention however barriers to implementation at the state level exist. Those involved in state-level policy making generally indicated a preference for Commonwealth-led regulation. This research suggests that implementation of regulation of the television marketing of unhealthy food to children should ideally occur under the direction of the Commonwealth government. However, given that regulation is technically feasible at the state level, in the absence of Commonwealth action, states/territories could act independently. The relevance of our findings is likely to extend beyond Australia as unhealthy food marketing to children is a global issue.

## Background

Overweight and obesity affects 23% of Australian children aged 2–16 years according to the most recent national survey [1]. Children′s food preferences and dietary intake have been linked to unhealthy food marketing [2]. Television advertising is the most significant means by which children are exposed to food marketing [3]. The majority of television food advertisements viewed by children are for foods high in sugar, salt or saturated fat [4]. Currently in Australia there is limited government regulation of the marketing of unhealthy food to children. Regulation is restricted to self-regulatory codes, devised and administered by the advertising and food industries [5].

In 2010 the National Preventative Health Taskforce called for government action to halt the effects of unhealthy food marketing on children′s health [6]. A recent analysis of a range of prevention interventions found regulation of unhealthy food marketing to be an effective and cost effective measure to address overweight and obesity in children [7]. Provisions exist for regulation of television food marketing to be introduced by the Commonwealth through their legislative power to make laws regarding postal, telegraphic, telephonic, and other like services, and by individual state and territory governments in Australia through their general power to pass legislation on locally relevant topics [5].

What is not yet well understood are the attitudes of those involved in the policy-making process towards regulatory intervention governing unhealthy food marketing. This paper reports on the perceptions of senior representatives from Australian state and territory governments, statutory authorities and non-government organisations regarding the regulation of unhealthy food marketing on television at the state level of government. An understanding of the views of those who contribute to the policy-making process enhances overall understanding of barriers and enablers to regulating the television marketing of unhealthy food to children in Australia.

## Methods

### Population and sample

The sample comprised senior representatives (including senior policy officers, directors and managers) with policy expertise or nutrition/physical activity expertise from state and territory government departments, statutory authorities and non-government organisations. Purposive sampling was employed to ensure a diverse range of stakeholders were consulted and an adequate reach across relevant government departments and each state and territory of Australia. Fifty-six participants were invited to participate via written letter and follow up telephone call. Forty-seven agreed to participate.

This paper focuses on a subset of data obtained from 47% of the participants (n=22) deemed to be content experts with regard to unhealthy food marketing. Content experts included representatives from health departments (n=12), central departments including treasury (n=2) and premier and cabinet (n=4), and relevant non-government organisations (n=4). Victoria was most highly represented with nine participants, four participants represented Western Australia, two each from Queensland, New South Wales and Tasmania, and one each from Northern Territory, Australian Capital Territory and South Australia.

### Instrumentation and data collection

An interview schedule was piloted and in-depth semi-structured interviews were conducted (JS) to elicit participants′ views of possible regulatory strategies for obesity prevention at the state level of government. Participants were asked to share their own ideas around regulatory interventions for obesity prevention before being asked to comment on a list of specific potential regulatory interventions, including the regulation of unhealthy food marketing to children. Semi-structured questioning was used to ascertain participants' views around feasibility including facilitators and barriers to implementing each of the regulatory interventions. Interviews with Victorian participants were conducted in person, while interstate participants were interviewed via telephone. Interviews were recorded and later transcribed verbatim. Mean interview duration was 62 minutes.

### Data analysis

Data analysis comprised three phases. Two researchers (JS & AC) independently coded the raw data. One researcher (AC) reviewed all codes and performed content analysis [8] to determine feasibility of unhealthy food marketing and thematic analysis [8] to collate categories and subsequently identify themes in the data. Three dominant themes and one rich point [9] were identified. Dominant themes included those to which more than a third of the sample contributed. The rich point comprised comments made by a small number of participants that added to the overall understanding of the feasibility of regulating unhealthy food marketing to children. The researcher (AC) conferred with fellow researchers (AP, JS) throughout the analysis to discuss interpretations. This enabled the interviewer (JS) to provide contextual information to researchers who were not present during the interviews [8].

### Ethics

Ethics approval was granted by Monash (2007-00-2150) and Deakin (EC 232–2007) Universities′ Human Research Ethics Committees and written informed consent was obtained from all participants.

## Results

Fourteen participants (64%) independently suggested regulation of television marketing of unhealthy food as a potential strategy for obesity prevention prior to commenting on the list of potential strategies provided by the research team. An additional six (27%) discussed regulation of television marketing of unhealthy food in response to the list of potential regulatory strategies for obesity prevention, while a further two (9%) made comments that were related to, but not specifically about, the regulation of unhealthy food marketing on television. Overall, the 20 participants who commented specifically about the regulation of television marketing of unhealthy food to children perceived it to be a feasible strategy.

### Themes

Three dominant themes emerged from the analysis: identification of regulation of television marketing as a Commonwealth, not state/territory, responsibility; the power of the food industry and; the need for clear evidence that demonstrates the effectiveness of regulation. A fourth theme - the need for evidence of community support for regulation - emerged as a rich point in the data. Table 1 outlines the themes, and categories that contributed to each theme.

**Table 1:** Themes and contributing categories

** Theme**	** Contributing categories**
Identification of regulation as a Commonwealth, not state, responsibility	·National approach required
	·National regulatory mechanisms already exist
	·Too complex for states to deal with
Food industry power	·Clever industry marketing
	·Generous marketing budgets
	·Food industry is politically strong
	·Need to work with industry
Evidence to support regulation	·Need evidence of cause and effect
	·Need evidence that regulation will be an effective strategy
Evidence of community support	·Community support for regulation
	·Community demand for action

Twenty participants discussed the feasibility of regulating television marketing of unhealthy food to children. Three (15%) participants deemed regulation of television marketing of unhealthy food at the state level to be feasible but did not elaborate to explain why. A further eight (40%) deemed regulation to be feasible providing certain limitations were addressed. Nine (45%) considered regulation of unhealthy food marketing at the state level to be unfeasible. Limitations and reasons for perceived unfeasibility are discussed in the dominant themes around the regulation of television marketing of unhealthy food.

### Identification of regulation as a commonwealth, not state, responsibility

Ten participants (50%) explicitly identified regulation of television marketing of unhealthy food as a Commonwealth responsibility. Reasons included the need for national consistency and leadership; the complexity of the regulation for individual jurisdictions; and the fact that national regulatory mechanisms already exist. Others identified that regulation of television marketing was possible by states and territories, yet stated that a national approach to regulation was most appropriate.

*…so while there is legal advice to say that on a technicality, broadcasting in certain viewing times, that state governments may have the ability to legislate - we’ve said that we think first and foremost a national approach is the best way to go…* (Department of Premier and Cabinet)

*…it′s got caught up with national policy platforms and no states or territories are likely to want to embarrass a national government that’s already said that they don’t wish to do this work.* (Health Department)

### Food industry power

Half of the participants identified that the food industry posed a substantial barrier to the regulation of television marketing of unhealthy food by government. Explanations included industries’ sizable marketing budgets and clever marketing strategies (indicative of their ability to get around any proposed regulation), as well as pressure against the government with regard to regulation.

*You sort of wonder if they start banning food advertising for kids whether* [fast food company] *will just be advertising toys and not saying anything about food because they′re always a step ahead of us.* (Health Department)

…*the fast food industry is such a big player, politically very strong…* (Health Department)

The idea of collaborating with industry rather than working against them was postulated but ways in which this could be done were not described.

*… it’s not getting into bed with the enemy, it’s about actually finding out what your enemy thinks.* (Health Department)

### Evidence to support regulation

Seven participants commented that further evidence was required to build a case for the regulation of television marketing of unhealthy food marketing. The reasons for needing more evidence were twofold. There was concern about the lack of evidence to support a causal relationship between exposure to unhealthy food marketing and childhood obesity.

*If there is strong evidence that children’s eating behaviour is somehow strongly linked to the advertising that they’re exposed to, then I think that we as a department would be able to mount a strong case…* (Department of Premier and Cabinet)

Furthermore, concern was expressed over a lack of evidence of effectiveness of the regulation itself; whether or not the regulation of unhealthy food marketing such as television advertisements would lead to a reduction in childhood obesity.

*I’d be interested to see if there was any sort of research that said that there could be some maximum benefits or achievement in terms of more fit and healthy children, lower costs, lower cost to government*. (Treasury Department)

### Evidence of community support

A rich point that emerged from the data arose from comments made by four participants (20%) who stated that community support was critical for governments to implement regulation of unhealthy food marketing to children. It was suggested that community views would need to be well understood before governments would consider regulating television marketing of unhealthy foods.

*If we don′t have community support for regulation and we go gung ho to push regulation and the community is not on board, we will lose, we′ll lose at the polls.* (Health Department)

*You can make a case for anything providing the community is with you.* (Health Department)

## Discussion

Overall, the regulation of television marketing of unhealthy food marketing to children was supported as a strategy for obesity prevention by those involved in state and territory policy-making processes in Australia. In general, participants commented that regulation should ideally occur at a Commonwealth level but there was some support for states/territories to regulate in the absence of Commonwealth action. The two other themes to emerge were the power of the food industry to block or find ways around regulatory approaches and the need for evidence to be presented to decision makers on the effects of marketing to children and the likely cost effectiveness of regulations to restrict such marketing. These findings are likely to have relevance to other countries which have a federal government system and a market driven economy similar to Australia as unhealthy food marketing to children is a global issue. The discussion below addresses each of the key themes that emerged from our research and considers the prospects for regulation of unhealthy food marketing in Australia.

### Identification of regulation as a commonwealth, not state, responsibility

Our research found that policy makers favour a national approach to the regulation of unhealthy food marketing, with very few enablers or solutions to implementation of such regulation by states and territories identified by the study participants. In the context that both the state and territory governments and the Commonwealth government in Australia have the potential to act to regulate unhealthy food marketing to children [10] this may imply a lack of political will due to the way policy-makers have framed the issue and suggests that a national approach to the regulation of unhealthy food marketing is more politically feasible.

The literature suggests Commonwealth-led regulation is likely to be the most efficient [11] and in Australia, the Obesity Policy Coalition recently outlined a legislative proposal that argues for comprehensive, national regulation of unhealthy food advertising to children [5]. Calls for a national approach to the issue are not limited to Australia. In the United States there is pressure for a national commitment and government action to address food marketing to children through the nutrition guidelines proposed by the Interagency Working Group for Foods Marketed to Children [12].

Beyond the preference for a national approach, the barriers to regulating television marketing of unhealthy food by states and territories, as identified by those involved in state and territory policy-making processes, do not appear to be intractable. Therefore, despite the evidence in favour of a national approach, should action not result at the Commonwealth level, willing states and territories could act independently. This could occur in collaboration or possibly through the actions of one particular state or territory, implementing regulation as a test case.

### Food industry power

Our analysis demonstrates that any approach to regulating television marketing of unhealthy food requires acknowledgement of barriers posed by the counter lobbying by the food industry. The food industry was perceived by participants as powerful because of their access to resources and their political influence. Despite this perception, specific examples of food industry exercising power or influence were not provided by participants. Our findings are consistent with research around stakeholders' views influencing the policy making process [13] and suggestions that stakeholders with business or economics interests tend to have the greatest influence over government [14]. Our findings suggest that food industry power is also likely to pose an issue in the case of Commonwealth-led regulation either by industry using their influence to inhibit the implementation of regulation, or by taking advantage of loopholes in regulation. Consequently, any regulation that is implemented needs to be comprehensive and expand on current self-regulatory codes which are limited in scope [4] and effectiveness [15,16], and restrict unhealthy food marketing through media and other avenues including, but not limited to, television [5].

### Evidence

Our research found that policy makers require scientific research evidence to demonstrate the effectiveness of regulating unhealthy food marketing. However, given that there is a body of existing research evidence demonstrating (i) that unhealthy food marketing influences children′s food preferences, purchase requests, and consumption behaviour [2,3], (ii) that restricting television food advertising is likely to contribute towards a reduction in obesity [17] and obesity prevalence [18], (iii) that regulation of unhealthy food marketing is a cost-effective obesity prevention strategy [6], and (iv) that self regulation has a minimal impact on the nutritional quality of television food advertisements targeted towards children [19], the nature of evidence sought by policy makers is unclear. Stakeholders frame problems and solutions differently and evidence can be sought to support a particular view, rather than inform a rounded view of an issue [20]. Given the wealth and breadth of evidence that exists, further work in this area should explore the extent to which this evidence is not reaching the relevant policy makers and the extent to which it is not deemed relevant for informing policy.

Our research also identified that evidence of community support is an important factor in the policy process, making the point that scientific research is just one type of evidence considered in the policy making process [21] and it is a broader view of evidence that comprises stakeholder views [13]. Organisational and community support for the regulation of unhealthy food marketing through advertising is evident. Numerous key public health agencies in Australia have expressed concern over marketing of unhealthy foods to children [5,6,22-24] and argue that current controls are both ineffective and inadequate [5,22,23]. Parents have expressed concern around unhealthy food marketing targeting children's vulnerabilities [25]. A recent national survey identified that 83% of consumers are in favour of government-led restrictions on television marketing of unhealthy food to children [26]. Once again, it will be important to explore whether this information is not reaching the relevant people, or whether the information itself is deemed not relevant.

While our study participants focussed on the Australian context, internationally there is increasing government support for action on unhealthy food marketing to children. Although self-regulation is still the favoured response by many governments, recent years have seen an increase in statutory regulation [27]. For example, in Norway and Sweden complete bans on food advertising to children under 12 years of age have been imposed [28], in the United Kingdom measures are in place to limit marketing of high fat and high sugar foods during children’s peak television viewing times [29], and in France food advertisements must include nutrition messages regardless of whether they are aimed at children or adults [27]. As governments around the world continue to respond to the issue of unhealthy food marketing to children, opportunities to study the impact of regulation arise, increasing the available evidence of the effect of regulation as a strategy for obesity prevention.

### Strengths and limitations

A strength of this study is that researchers spoke directly with a broad range of senior people involved in the policy-making process. This allowed for analysis of a range of viewpoints across states and territories and various sectors and government departments. A limitation however is that the cohort of 47 survey participants provides insight into the views of only a sample of those involved in the policy making process. Furthermore, the initial sample selection and researchers' definition of content experts from this cohort may have unintentionally excluded others involved in the policy making process, limiting the depth of discussion. Finally, the data used for this discussion came from interview questions designed for a broader study exploring state government regulatory approaches for promoting healthy food system and physical activity environments, limiting the potential for in-depth discussions of the feasibility of regulating television marketing of unhealthy food to children.

## Conclusion

This research indicates that those directly involved in the policy-making process in Australia perceive the best approach to regulating the marketing of unhealthy food to children is via a Commonwealth-led approach. If this does not occur, the research does not suggest that there are any intractable technical barriers to regulating television marketing of unhealthy food at a state level. However significant political barriers have been identified. Implementation of regulations to reduce advertising of unhealthy food to children is likely to require concerted efforts on a number of fronts, including reaching agreement for action between states and Commonwealth; balancing the health agenda against the power of the food industry; and communicating the outcomes of such regulation in terms of benefits and costs deemed relevant by those involved in the policy-making process.

## Competing interests

The authors declare that they have no competing interests.

## Authors’ contributions

AP, BL, BS and ML conceived of the research project within which this work sits. JS and AP conceived this study. AC executed and designed the study protocol, performed the analyses and wrote the article. JS, HM, BC and GS contributed to the data collection and analysis. SA contributed to the study design. All authors read and approved the final manuscript.

## Pre-publication history

The pre-publication history for this paper can be accessed here:

http://www.biomedcentral.com/1471-2458/12/1123/prepub
